# GidA, a tRNA Modification Enzyme, Contributes to the Growth, and Virulence of *Streptococcus suis* Serotype 2

**DOI:** 10.3389/fcimb.2016.00044

**Published:** 2016-04-19

**Authors:** Ting Gao, Meifang Tan, Wanquan Liu, Chunyan Zhang, Tengfei Zhang, Linlin Zheng, Jiawen Zhu, Lu Li, Rui Zhou

**Affiliations:** ^1^State Key Laboratory of Agricultural Microbiology, College of Veterinary Medicine, Huazhong Agricultural UniversityWuhan, China; ^2^Veterinary Medicine Laboratory, Institute of Animal Husbandry and Veterinary Science, Hubei Academy of Agricultural ScienceWuhan, China; ^3^Wuhan Chopper Biology Co., Ltd.Wuhan, China; ^4^Cooperative Innovation Center of Sustainable Pig ProductionWuhan, China

**Keywords:** *Streptococcus suis* (*S. suis*), glucose-inhibited division protein (GidA), tRNA modification, iTRAQ, growth, capsule synthesis, virulence

## Abstract

Glucose-inhibited division protein (GidA), is a tRNA modification enzyme functioning together with MnmE in the addition of a carboxymethylaminomethyl group to position 5 of the anticodon wobble uridine of tRNA. Here, we report a GidA homolog from a Chinese isolate SC-19 of the zoonotic *Streptococcus suis* serotype 2 (SS2). *gidA* disruption led to a defective growth, increased capsule thickness, and reduced hemolytic activity. Moreover, the *gidA* deletion mutant (Δ*gidA*) displayed reduced mortality and bacterial loads in mice, reduced ability of adhesion to and invasion in epithelial cells, and increased sensitivity to phagocytosis. The iTRAQ analysis identified 372 differentially expressed (182 up- and 190 down-regulated) proteins in Δ*gidA* and SC-19. Numerous DNA replication, cell division, and virulence associated proteins were downregulated, whereas many capsule synthesis enzymes were upregulated by *gidA* disruption. This is consistent with the phenotypes of the mutant. Thus, GidA is a translational regulator that plays an important role in the growth, cell division, capsule biosynthesis, and virulence of SS2. Our findings provide new insight into the regulatory function of GidA in bacterial pathogens.

## Introduction

*Streptococcus suis* is an important zoonotic pathogen causing lethal infections in humans and pigs (Lun et al., 2007). *S. suis* infection in human is an emerging public health issue, whereas that in pigs causes severe economic problems in the pig industry (Wertheim et al., 2009). Two large outbreak of human *S. suis* infections were reported in China in 1998 and 2005, resulting in 229 infections and 52 deaths (Yu et al., 2006; Lun et al., 2007). Among the 33 serotypes classified on the basis of antigenicity of capsular polysaccharide (CPS), *S. suis* serotype 2 (SS2) is the most virulent and prevalent strain isolated from diseased pigs (Smith et al., 1999). Several virulence-associated factors responsible for the pathogenecity of *S. suis*, such as muramidase-released protein, suilysin (Sly), extracellular factor, fibrinonectin- and fibrinogen-binding proteins, enolase, arginine deiminase system (ADS), and glyceraldehyde-3-phosphate dehydrogenase (GAPDH), were identified over the past decade (Jing et al., 2008; Feng et al., 2014).

*S. suis* infection is a major cause of meningitis, septicemia, and arthritis. *S. suis* primarily colonizes the palatine tonsils, which is one of its natural habitats, then breaches epithelial cell barriers, reaches the bloodstream, disseminates through the blood circulation system, and finally invades different organs of the host (Fittipaldi et al., 2012). In this process, many proteins are regulated (up-regulated or down-regulated) at the translation level in response to surroundings change and environmental signals. However, the regulatory mechanism of genes which are preferentially regulated by this pathogen during specific stages of host infection has not yet been clearly demonstrated. GidA-like proteins, functioning as a tRNA modification enzyme, are widely distributed in nature and conserved among eukaryotes and prokaryotes (Yim et al., 2006). GidA is a FAD-binding protein and, together with MnmE, catalyzes the addition of carboxymethylaminomethyl group at position 5 of the wobble uridine of tRNAs (Shi et al., 2009). This modification contributes to proper and efficient protein translation (Fislage et al., 2014). GidA and MnmE serve essentially to prevent premature translation termination resulting from (+2) translational frameshifts (Brégeon et al., 2001). GidA protein plays a different role in many bacteria: in *Escherichia coli*, deletion of *gidA* affects cell division when it is grown on glucose (Von Meyenburg et al., 1982); in *Streptococcus mutans, gidA* is involved in survival under stress conditions (Li et al., 2014); in *Aeromonas hydrophila, gidA* regulates virulence protein, cytotoxic enterotoxin (Sha et al., 2004); in *Salmonella enterica*, disruption of *gidA* affects cell division and regulates the virulence proteins (Shippy et al., 2012; Rehl et al., 2013); and in *Pseudomonas syringae, gidA* is a global regulator (Kinscherf and Willis, 2002). In fact, GidA can regulate the expression of multiple proteins at the level of translation through tRNA modification (Kinscherf and Willis, 2002; Yim et al., 2006), and thus can regulate the survival of bacteria under stress condition in response to environmental signals.

In our previous study, the *gidA* gene was identified to be up-regulated in the brains and lungs of *S. suis* infected pigs, suggesting that GidA may play a role during the infection (Li et al., 2010). In this study, we identified a functional gene SSU05_2163, which is annotated as *gidA* in *S. suis* 05ZYH33 genome and encodes a tRNA modification enzyme. Our data demonstrate that GidA is a translational regulator that affects cell growth and division, capsule polysaccharide biosynthesis, and expression of virulence proteins in *S.suis*.

## Materials and methods

### Bacterial strains, plasmids, and growth conditions

The bacterial strains and plasmids used in this study are listed in Table 1. The SS2 strain SC-19 was isolated from a sick pig during an epidemic outbreak in Sichuan Province in China in 2005 (Li et al., 2009). Bacterial solution for experiment was prepared as follows: SC-19 and Δ*gidA* were grown at 37°C either in tryptic soy broth, or on tryptone soy agar (Difco, France) containing 10% fetal bovine serum (Sijiqing, Hanzhou, China), and then the cultures were centrifuged and washed thrice with saline. To be consistent with the isobaric tags for relative and absolute quantitation (iTRAQ) analysis, all of the bacterial pellets were resuspended in the chemically defined medium (CDM) (Van De Rijn and Kessler, 1980) supplemented with 1% glucose and grown to mid-log phase at 37°C. *E. coli* DH5α was used as host strain for cloning and maintained in Luria-Bertani (LB) broth (Difco) or plated on LB agar at 37°C. The antibiotic concentration prepared for wild-type SC-19 was 20 μg/ml streptomycin. The antibiotic concentrations used to select the mutant strain Δ*gidA* were 100 μg/ml for spectinomycin and 90 μg/ml for erythromycin. The kanamycin concentration used to select *E. coli* strains harboring the plasmid of interest was 25 μg/ml.

**Table 1:** **Summary of bacterial strains and plasmid used in this study**.

**Strain or plasmid**	**Characteristics and function^a^**	**Sources or references**
**BACTERIAL STRAINS**		
SC-19	*S. suis* serotype 2, wide- type (Strep ^r^)	Li et al., 2009
*ΔgidA*	SC-19 *gidA*::*erm* (Strep ^r^ Erm ^r^)	This study
*E. coli* DH5α	Cloning host for recombinant vector	Trans
**PLASMID**		
pAT18	Contains an Erm ^r^ gene expressing erythromycin resistance rRNA methylase	Trieu-Cuot et al., 1991
pET28a	Expression vector; Kan ^r^	Novagen
pSET4s	*E. coli*- *S. suis* shuttle vector; Spc ^r^	Takamatsu et al., 2001
pSET4s-G	Derived from pSET4s used to knock out *gidA* in SC-19; Spc ^r^ Erm ^r^	This study

a *Strep ^r^, streptomycin resistant; Erm ^r^, erythromycin resistant; Kan ^r^, kanamycin; Spc ^r^, spectinomycin, resistant*.

### Knockout of *gidA*

To construct a Δ*gidA* mutant strain, we used the thermosensitive suicide vector pSET4s to delete *gidA* through homologous recombination as previously described (Takamatsu et al., 2001). Primers used in this study were designed according to the genome sequence of *S. suis* 05ZYH33 (GenBank accession number CP000407; Table 2). Two pairs of specific primers, Gup-F/Gup-R and Gdown-F/Gdown-R were used to clone the *gidA* upstream and downstream of the homologous regions into pSET4s. The *erm*^*r*^ expression cassette was amplified from pAT18 by using primers Erm-F/Erm-R and then inserted between the upstream and downstream homologous arms in the recombinant pSET4s to generate the *gidA*-knockout vector pSET4s-G.

**Table 2:** **Primers used for PCR amplification and detection**.

**Primers**	**Primers sequence (5′−3′)^a^**	**Amplification for**
Gup-F	CTTCAAGCTTGCTTTTGTGGACTTA	Upstream border of *gidA*
Gup-R	GTTTGTCGACTCATGTTGTTCTCTCCT	
Gdown-F	GATCCCGGGGGCTGTTCTTTTCGC	Downstream border of *gidA*
Gdown-R	CCCCGAATTCTTCCTTGACCACAACC	
Erm-F	GTCTGGATCCCTTAGAAGCAAACTTAA	Erm ^r^ gene
Erm-R	GTTAGGATCCATCGATACAAATTCCCCG	
GidA-F	CGGGATCCATGACACACACATTTGCAGA	*gidA* gene
GidA-R	CGCTCGAGTTAGTGACTGTCCTTTGATTT	
2162-F	GTGATGAAAAGATTTCGATT	Downstream gene of *gidA*
2162-R	TTATCCAAAGTCAAGCCA	
2164-F	GGTTGATTATAAAAGATGG	Upstream gene of *gidA*
2164-R	TCATGTTGTTCTCTCCTT	

a *Underlined nucleotides denote enzyme restriction sites*.

To obtain isogenic mutant Δ*gidA*, we electro transformed pSET4s-G plasmid into SC-19 competent cells (Zhang et al., 2012). The mutant strain was screened on TSA plates owing to its sensitivity to spectinomycin and resistance to erythromycin. To confirm the mutant, we amplified *gidA* through PCR by using the primers GidA-F/GidA-R.

### RNA extraction and RT-PCR

To confirm the mutant strain Δ*gidA*, we performed RT-PCR according to our previously reported methods (Tan et al., 2015). Briefly, RNA was isolated using SV Total RNA Isolation System (Promega, USA) according to the manufacturer's instructions. In addition, cDNA was synthesized using HiScript Q Select RT SuperMix (Vazyme, China) according to the manufacturer's instructions.

To confirm whether the upstream and downstream genes of *gidA* are unaffected and functioning normally, we designed the primers of SSU05_2162, *gidA*, and SSU05_2164 for RT-PCR (Table 2) from the cDNA.

### Western blot

To further confirm the mutant strain Δ*gidA*, we performed Western blot analysis according to our previously reported methods (Tan et al., 2015). Mouse anti-GidA serum was produced as described previously (Li et al., 2011) by using recombinant GidA protein. The PVDF membranes (Invitrogen, USA) were probed with primary antibodies against GidA (1:1000) or 3-phosphoglycerate kinase (PGK) (1:5000; Invitrogen) (Zhang et al., 2014). After washing, the membranes were incubated with goat anti-mouse IgG (H+L)-HPR (1:5000; Southern Biotech, USA). Antibody-tagged protein bands were detected by using Western ECL Substrate Kit (Bio-Rad, USA).

### Transmission electron microscopy (TEM)

To obtain an overview of the morphology of SC-19 and Δ*gidA*, we performed TEM as described previously (Zheng et al., 2011). The samples grown in CDM were harvested at mid-log phase and fixed with 2.5% glutaraldehyde overnight. The samples were then treated with 2% osmium tetroxide for 2 h and dehydrated in a serial dilution of ethanol. The dehydrated cells were embedded in epoxy resin and cell morphology was observed using an H-7650 TEM (HITACHI, Ltd., Tokyo, Japan). 20 bacterial cells were randomly chosen from the TEM micrographs to measure the thickness of capsule by using the software Image J, and then statistically analyzed on GraphPad prism 5.

### Hemolysin assay

Hemolysin activity was tested as described previously (Jacobs et al., 1994) with some modifications. Briefly, *S. suis* strains were grown in CDM up to mid-log phase, and the culture supernatant was collected by centrifugation at 12000 g for 2 min. The test samples (100 μl) were incubated with 2% sheep erythrocyte suspension (100 μl) in saline for 2 h at 37°C and CDM was used as negative control. Unlysed erythrocytes were centrifuged at 1500 g for 15 min, and 100 μl supernatant was transferred into a new plate (Jet Biofil, China). Absorption was subsequently measured at 550 nm by using a microELISA reader (Biotek, Vermont, USA).

### Mouse infection experiments

This study was performed in accordance with the Guide for the Care and Use of Laboratory Animals Monitoring Committee of Hubei Province, China, and the protocol was approved by the Committee on the Ethics of Animal Experiments of the College of Veterinary Medicine, Huazhong Agricultural University. All efforts were made to minimize the suffering of the animals used in the study.

To detect the role of GidA in *S. suis* virulence, we divided 30 6-week-old female specific-pathogen-free (SPF) Kun-Ming mice into three groups (10 mice per group). Groups 1 and 2 were inoculated via intraperitoneal injection with 3 × 10^9^ CFU of either SC-19 (the LD_50_ for mice is 1.5 × 10^9^ CFU) or Δ*gidA*. Saline was applied in Group 3 as negative control. The mice were observed for 7 days to obtain steady survival curves.

To detect the role of GidA on colonization in different organs, we performed murine colonization assay as described previously (Marion et al., 2011). A total of 15 6-week-old female SPF Kun-Ming mice were inoculated intraperitoneally with 1 × 10^8^ CFU of a 1:1 mixture of mid-log phase SC-19 and Δ*gidA*. Saline was applied as negative control in five mice. At 12 h, 1 day, and 3 days post infection (dpi), brain, lung, and spleen were obtained from five mice. The samples were homogenized after weighing, and serial dilutions were plated onto TSA agar. To count the colonies, we used 20 μg/ml streptomycin for SC-19, whereas 20 μg/ml streptomycin and 90 μg/ml erythromycin were used for Δ*gidA*.

### Phagocytosis assay

To probe the resistance of each strain to phagocytosis, we performed an experiment as described previously (Li et al., 2013). RAW264.7 cells were scraped up and resuspended in antibiotics-free medium. After adhering to six-well cell culture plate (Falcon, USA), the cells were infected with SS2 at mid-log phase to reach a ratio of 10 bacteria per macrophage (MOI = 10:1). Phagocytosis proceeded for 30 min at 37°C and then the cells were washed with PBS thrice. The cells were incubated in medium containing penicillin (100 μg/ml) for 1 h at 37°C to kill extracellular bacteria. The culture supernatant was plated on TSA plates to confirm whether the antibiotics effectively killed the extracellular bacteria. The macrophages were then lysed in 1 ml of sterile distilled water. Viable intracellular bacteria were determined by plating a serial dilution of the lysates on TSA agar.

### Adhesion and invasion assays

To evaluate the adhesion and invasion capacity of each strain, we performed an experiment as described previously (Ferrando et al., 2014). For the adherence assay, HEp-2 cells were infected with SS2 at mid-log phase to reach a ratio of 100 bacteria per cell (MOI = 100:1) and then incubated for 30 min at 37°C.The monolayers were washed with PBS thrice and lysed in 1 ml of sterile distilled water. Adherent bacteria (cell-associated bacteria) were determined by plating a serial dilution of the lysates on TSA agar. For invasion assay, the cells were incubated with bacteria for 2 h to allow invasion. The cells were subsequently incubated in medium containing penicillin (100 μg/ml) for 2 h to kill extracellular and surface-adherent bacteria. The monolayers were washed with PBS thrice and lysed in 1 ml of sterile distilled water. Invasive bacteria (intracellular bacteria) were determined by plating a serial dilution of the lysates on TSA agar.

### Protein extraction, digestion, and labeling with iTRAQ reagents

SC-19 and Δ*gidA* cells at mid-log phase were cultured in CDM as described above. Three independent biological replicates were homogenized in liquid nitrogen and then precipitated using trichloroacetic acid and acetone. The pellets were suspended in lysis buffer (4% SDS, 100 Tris-HCl, and 1 mMDTT; pH7.6) and heated for 10 min at 100°C. The cell suspensions were sonicated for 5 min (10 s sonication with 15 s interval) on ice and then protein concentration in supernatants was determined through Bradford protein assay. Each sample (200 μg) was digested with 3 μg of trypsin (Sigma, USA) at 37°C for 16 h. iTRAQ labeling was performed according to the manufacturer's protocol (Applied Biosystems, Foster City, CA, USA). Briefly, each iTRAQ reagent was dissolved in 70 μl of ethanol and added into the peptide mixture, respectively. After incubation for 2 h at room temperature, the reaction was quenched by adding 0.5% formic acid. iTRAQ tags were labeled as follows: the three SC-19 samples were labeled with iTRAQ 114, iTRAQ 115, and iTRAQ 116; and the three Δ*gidA* samples were labeled with iTRAQ 117, iTRAQ 118, and iTRAQ 119. The labeled peptides were combined and fractionated by using strong cation exchange (SCX) chromatography.

### LC-MS/MS analysis

After separation by SCX chromatography on an AKTA purifier 100 (GE Healthcare, USA), equal amounts of digested protein were loaded into a Thermo Scientific EASY column(2 cm^*^100 μm 5 μm-C18) and then washed with solvent A (99% H_2_O, and 0.1% formic acid). By applying solvent B (84% acetonitrile, 16% H_2_O, and 0.1% formic acid), the peptides were eluted from the trapping column over a Thermo scientific EASY column (75 μm^*^100 mm 3 μm-C18) with a gradient (0–45% B for 100 min at 250 nl/min, 35–100% B for 8 min, 100% B for 12 min) using Thermo scientific Easy nLC system. MS/MS was carried out with a Q-Exactive mass spectrometer (Thermo Finnigan, USA) setting in a positive ion mode and data-dependent manner choosing the most abundant precursor ions with a full MS scan from 300 to 1800 *m/z*, resolution of 70,000 at *m/z* 200. Determination of the target value was based on automatic gain control (AGC). Dynamic exclusion duration was 40 s. MS/MS scan was acquired at a resolution of 17,500 at *m/z* 200. Normalized collision energy was 30 eV and the under fill ratio was set at 0.1%. Quantitation achieved by comparison of the peak areas and resultant peak ratios for either four MS/MS reporter ions, which range from 114 to 117 Da, or eight MS/MS reporter ions, which range from 113–119 to 121 Da.

### Proteomic data analysis

The acquired raw MS/MS data files were processed by Proteome Discoverer 1.4 (Thermo Scientific, USA) and searched by Mascot 2.2 (Matrix Science, Boston, MA) against the uniprot_*Streptococcus*_*suis*_23318_20150708.fasta (23,318 sequence, downloaded July 8, 2015). The search was conducted by applying trysin as a specific enzyme and the parameters used for normal peptides were as follows: peptide mass tolerance: 20 ppm, fragment mass tolerance: 0.1 Da, max missed cleavages: 2, fixed modifications: carbamidomethyl (C), iTRAQ8plex(K), and iTRAQ8plex(N-term), variable modifications: oxidation (M), database pattern: decoy, false-discovery rate (FDR) ≤ 0.01 (Sandberg et al., 2012). Each of the confident protein identification involved at least one unique peptide. The quantitative protein ratios were weighed and normalized by the protein median ratio in Mascot. To evaluate the differentially expressed (DE) proteins between Δ*gidA* and SC-19, we use the fold change of >1.2 or < 0.8333 and FDR of < 0.05 to represent up- or down-regulation.

### Statistical analysis

Unless otherwise specified, the data were analyzed using two-tailed, unpaired *t*-tests and all experiments were performed in triplicate at least thrice. All of the data were expressed as mean standard errors of the means (SEM), and *p* < 0.05 is the threshold for significance. Statistical analysis was performed on GraphPad prism 5.

## Results

### Construction and characterization of *ΔgidA*

**Figure 1:**
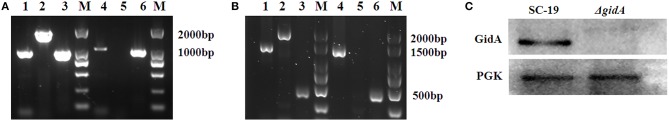
**Confirmation of the isogenic mutant Δ*gidA*. (A)** Combined PCR analyses of the Δ*gidA* mutant. Lanes 1 and 4 represent the amplification of the upstream border of *gidA* using the primer set Gup-F and Gup-R. Lanes 2 and 5 represent the amplification of *gidA* using the primer set GidA-F and GidA-R. Lanes 3 and 6 represent the amplification of the downstream border of *gidA* using the primer set Gdown-F and Gdown-R. Lanes 1–3 use genomic DNA of SC-19 as templates, whereas Lanes 4–6 use genomic DNA of Δ*gidA* as templates. **(B)** Confirmation of the Δ*gidA* mutant by RT-PCR. Lanes 1 and 4 represent the amplification of downstream gene of *gidA* using the primer set 2162-F and 2162-R. Lanes 2 and 5 represent the amplification of *gidA* using primer set GidA-F and GidA-R. Lanes 3 and 6 represent the amplification of upstream gene of *gidA* using the primer set 2164-F and 2164-R. Lanes 1–3 use cDNA of SC-19 as templates, whereas Lanes 4–6 use cDNA of Δ*gidA* as templates. **(C)** Confirmation of the Δ*gidA* mutant by Western blot analysis. The supernatant of cell lysate from SC-19 and Δ*gidA* was disposed for immunoblot analysis with GidA or PGK polyclonal antibodies. An antibody directed against PGK was used as loading control.

**Figure 2:**
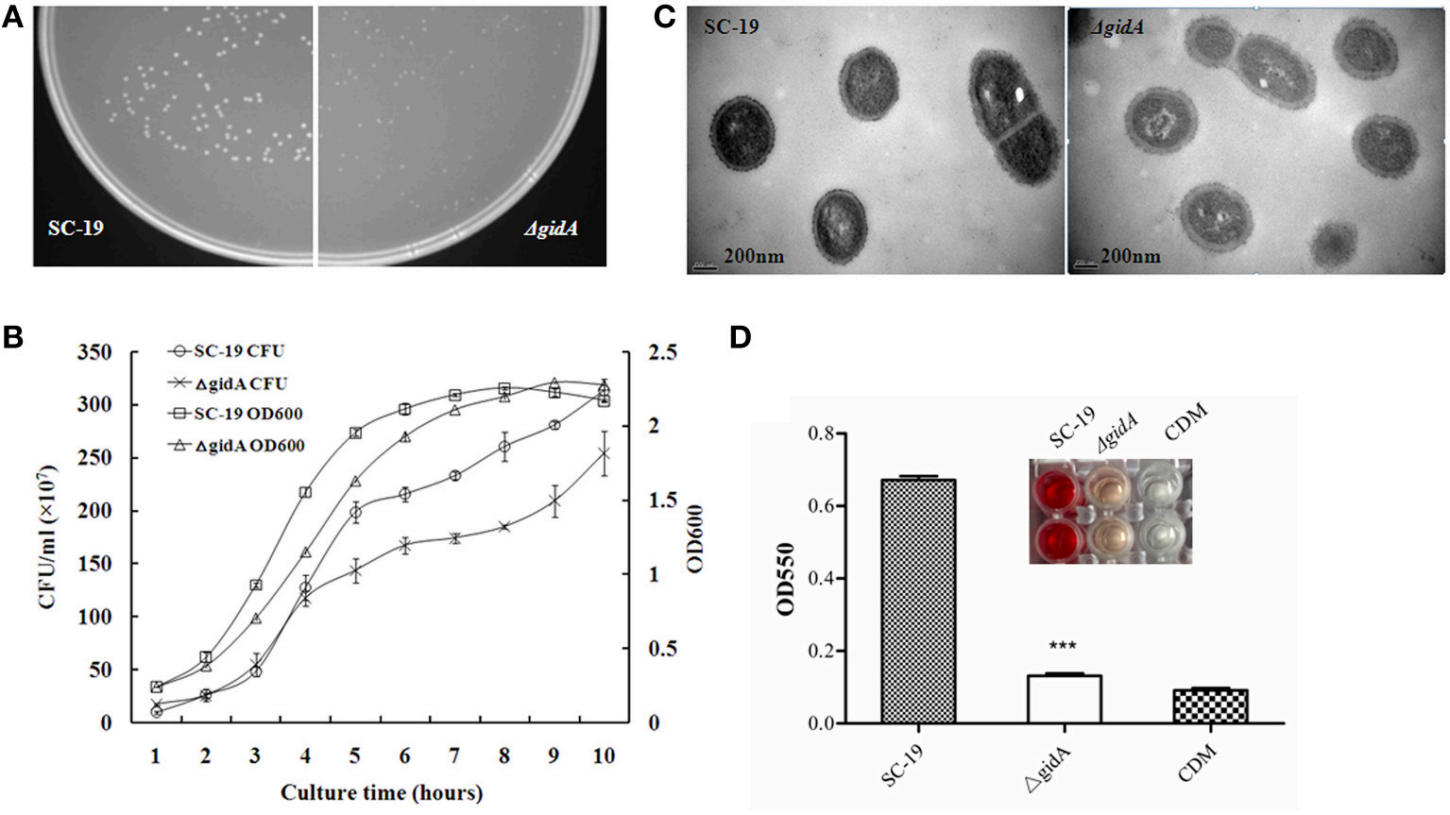
**Characterization of *gidA* mutant. (A)** SC-19 and Δ*gidA* mutant cultured overnight at 37°C on TSA plates. **(B)** Bacterial cell density was measured spectrometrically at 600 nm, and separate aliquots of the bacterial suspensions were serially diluted and plated to determine CFU numbers per milliliter. Data were collected at the indicated times. **(C)** Transmission electron micrographs of bacteria; the bars represent 400 nm (^***^p < 0.001). **(D)** Microplate showing hemolytic activity of the supernatants collected from SC-19 and Δ*gidA* mutant grown in CDM. Absorption was measured at 550 nm to determine suilysin production (^***^p < 0.001). CDM was used as negative control.

The colonies sensitive to spectinomycin and resistant to erythromycin were selected as candidates of *gidA* deletion mutants, which were confirmed by PCR (Figure 1A), RT-PCR (Figure 1B), and Western blot analysis (Figure 1C). The colonies of Δ*gidA* appeared smaller than those of SC-19 when cultured on TSA plates overnight (Figure 2A). The growth curves showed that Δ*gidA* grew slower in the CDM than SC-19 (Figure 2B). However, no obvious difference in CFU counts was observed during the initial 3 h of growth. TEM revealed that the mean capsule was significantly thicker in Δ*gidA* (118 ± 5 nm) than in SC-19 (54 ± 3 nm; *p* < 0.001; Figure 2C).

### Reduced hemolytic activity

The hemolysin assay showed that the hemolytic activity of Δ*gidA* was significantly reduced compared to that of SC-19 (Figure 2D), indicating that GidA could regulate the hemolytic activity of *S. suis*.

### Attenuated virulence and decreased bacterial loads in mice

**Figure 3:**
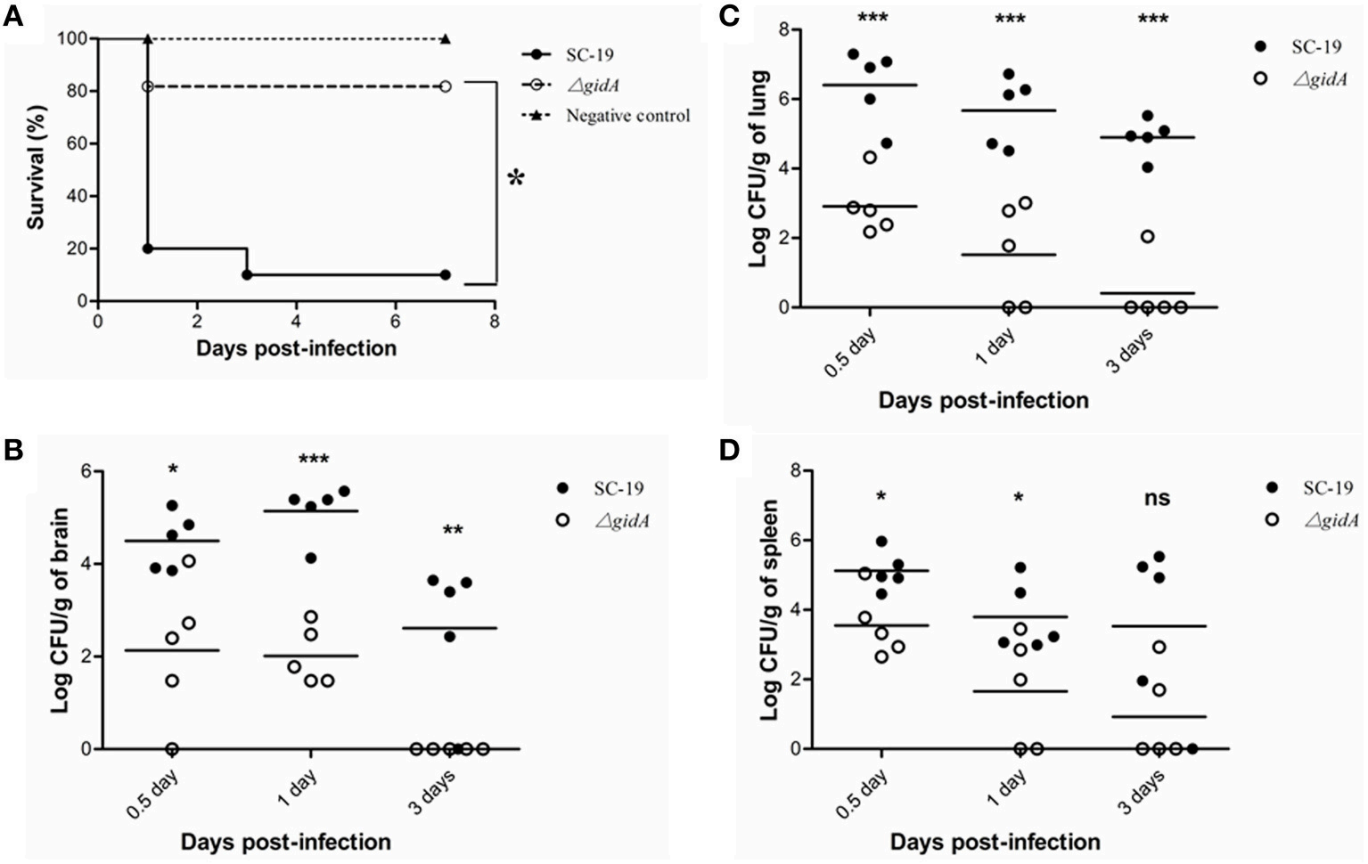
**Mouse infection experiments. (A)** Survival curves for mice in experiment infection. Ten mice in each group were separately injected intraperitoneally i with 3 × 10^9^ CFU/mice of SC-19 and Δ*gidA*. Ten mice were inoculated with saline and served as negative control. Significant difference in survival between different groups were analyzed by Log Rank test (*p* < 0.05). **(B)** Bacteria loads in **(B)** brain, **(C)** lung, and **(D)** in spleen. The SC-19 and Δ*gidA* mutant strains were distinguished by erythromycin added in the TSA plates. Statistical significance was determined by two-tailed *t*-test (ns, *p* > 0.05; ^*^p < 0.05; ^**^p < 0.01; ^***^p < 0.001).

Mice were experimentally infected to detect the role of *gidA* in *S. suis* virulence. All of the SC-19-infected mice displayed severe clinical symptoms, such as septicemia and meningitis during 1 dpi, and most of the infected mice (9/10) died during the 7 day observation period. By contrast, the Δ*gidA*-infected mice exhibited more slight clinical symptoms and low mortality (2/10) (Figure 3A). Therefore, Δ*gidA* virulence is markedly attenuated.

To better evaluate the pathogenecity of Δ*gidA*, we performed a colonization experiment by using intraperitoneal route of inoculation. Bacteria were recovered from brains, lungs, and spleens at different time points post infection. The bacterial loads in brain, lung, and spleen were much lower in Δ*gidA* than SC-19 from 12 h to 3 dpi, and the mutant strain was almost cleared at the 3 dpi (Figures 3B–D).

### Greater sensitivity to phagocytosis by RAW264.7 cells

**Figure 4:**
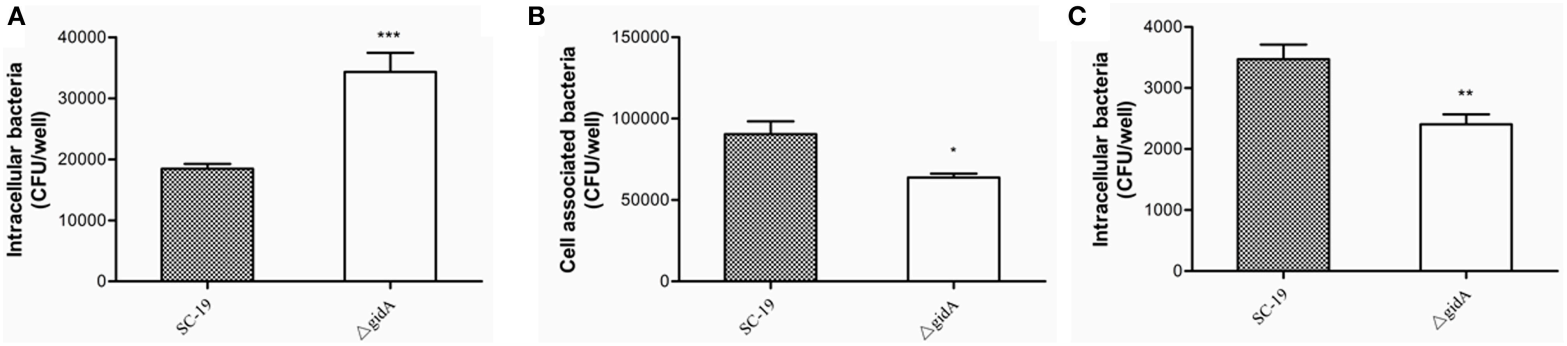
**Phagocytosis, adhesion, and invasion assays. (A)** Phagocytosis of *S. suis* by murine macrophages. SC-19 and Δ*gidA* mutant were incubated with RAW264.7 cells for 30 min at a multiplicity of infection of 10 (MOI = 10:1). Penicillin (100 μg/ml) was then used to kill the extracellular bacteria. The mutant strain Δ*gidA* showed significantly reduced anti-phagocytosis capability compared with SC-19 (^***^p < 0.001). **(B)** The mutant strain Δ*gidA* showed significantly reduced levels of adherence to HEp-2 cells compared with the = degree of adherence of SC-19 (^*^p < 0.05). **(C)** The mutant strain Δ*gidA* showed significantly reduced levels of invasion of HEp-2 cells compared with that of SC-19 (^**^p < 0.01).

To investigate the role of *gidA* on phagocytosis of *S. suis*, we performed a phagocytosis assay by using RAW264.7 cells. The numbers of intracellular bacteria for Δ*gidA* (34,320 ± 3130 CFU/well) were approximately two fold higher than those of SC-19 (18430 ± 821 CFU/well; *p* < 0.001; Figure 4A). This result indicates that inactivation of *gidA* can impair the capacity of *S.suis* to resist phagocytosis by macrophages.

### Impaired adhesion and invasion capability to epithelial cells

The adhesion assay revealed that the numbers of cell-associated bacteria of Δ*gidA* (63,800 ± 2437 CFU/well) were significantly lower than those of SC-19 (90,400± 7891 CFU/well; *p* < 0.05; Figure 4B). In the invasion assay, the numbers of the cell intracellular bacteria of Δ*gidA* (2406 ± 163 CFU/well) were significantly lower than those of SC-19 (3470 ± 241 CFU/well; *p* < 0.01; Figure 4C). These results suggest that deletion of *gidA* impairs the ability of *S. suis* to adhere to and invade in epithelial cells.

### Analysis of the DE proteins

The iTRAQ labeling was used to identify the DE proteins in SC-19 and Δ*gidA.* A total of 1449 proteins were detected and quantified, 372 of which were DE proteins, including 182 up-regulated and 190 down-regulated proteins (Table S1).

**Figure 5:**
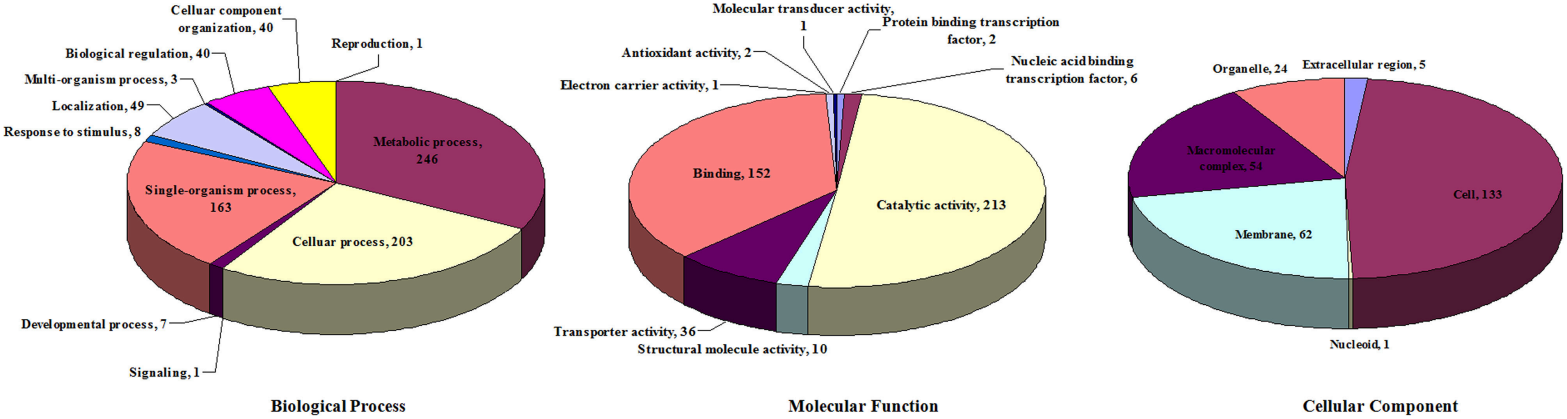
**Distribution of differentially expressed proteins according to GO annotation**.

These DE proteins were annotated using Blast2GO according to biological process, molecular function, and cellular component (Figure 5). In terms of biological process, the 372 DE proteins were classified into 11 categories. The top categories with the highest number of DE proteins were metabolic process (246, 66.1%), cellular process (203, 54.6%), and single-organism process (163, 43.8%); these three functional categories are the most important in *S. suis* response to environmental stresses. In terms of molecular function, the 372 DE proteins were classified into nine categories. The top two categories with the highest number of DE proteins were catalytic activity (213, 57.3%) and binding (152, 40.9%). In terms of cellular component, the 372 DE proteins were classified into six categories. The top three categories with the highest number of DE proteins were cell (133, 35.8%), membrane (62, 16.7%), and macromolecular complex (54, 14.5%).

#### Proteins involved in growth and cell division

Many growth- and cell division-associated proteins were regulated in the mutant strain (Table 3). Among these DE proteins, all of the DNA replication-, recombination- and repair-related proteins, including DNA primase RnmV, DNA gyrase (GyrA and GyrB), superfamily I DNA/RNA helicase (PcrA), site-specific recombinases (XerS andXerD), DNA repair ATPase (RecN), DNA recombination protein (RmuC), ribonucleases (RnhB, RNase H, G, and E), and 3-methyladenine DNA glycosylase (Tag), were down-regulated. The other DE proteins, including DivIVA, FtsQ, FtsX, FtsI, GpsB, StpK, PhpP, Cps2C, and MurD, are involved in cell division. Except for FtsX and GpsB, all other proteins were down-regulated.

**Table 3:** **Differentially expressed proteins associated with cell growth and division, capsule synthesis, and virulence**.

**Protein name**	**Locus**	**Functions**	**Ratio(*ΔgidA*/SC-19)**	**Peptides**	**Sequence coverage (%)**
**CELL GROWTH AND DIVISION**					
Cps2C	SSU05_0566	Tyrosine-protein kinase Wze	0.4555	1	28.57
DivIVA	SSU05_0487	Cell division initiation protein	0.8229	1	59.83
–	SSU05_0133	Adenine-specific DNA methylase	0.8010	8	34.70
FtsI	SSU05_1354	Cell division protein	0.8185	5	31.84
FtsQ	SSU05_0478	Cell division septal protein	0.8223	8	28.33
FtsX	SSU05_1410	Cell division protein	1.2542	6	24.60
GpsB	SSU05_0417	Cell division protein	1.3016	6	62.16
GyrA	SSU05_1267	DNA gyrase subunit A	0.8140	9	16.43
GyrB	SSU05_1510	DNA gyrase subunit B	0.7152	2	45.38
MurD	SSU05_0476	UDP-*N*-acetylmuramoyl-L-alanyl-D-glutamate synthetase	0.7718	14	39.87
PcrA	SSU05_0731	Superfamily I DNA/RNA helicase	0.8044	15	20.42
PhpP	SSU05_0427	Serine/threonine protein phosphatase	0.8067	11	59.59
RecN	SSU05_1651	DNA repair ATPase	0.8321	14	35.99
RmuC	SSU05_1991	DNA recombination protein	0.7751	8	19.90
RnhB	SSU05_0996	Ribonuclease HII	0.8074	8	35.41
RnmV	SSU05_2010	DNA primase	0.7144	6	26.53
RNase H	SSU05_0226	Ribonuclease HIII	0.7297	4	14.86
StkP	SSU05_0428	Serine/threonine protein kinase	0.8129	5	32.53
Tag	SSU05_0061	3-methyladenine DNA glycosylase	0.7738	1	10.58
–	SSU05_0872	Chromosome segregation ATPase	0.7753	13	25.46
XerS	SSU05_0881	Site-specific tyrosine recombinase	0.6361	1	5.90
XerD	SSU05_1702	Site-specific tyrosine recombinase XerD-like protein	0.5529	3	15.64
–	SSU05_1815	Ribonucleases G and E	0.6327	6	6.42
**CAPSULE SYNTHESIS**					
Cps2C	SSU05_0566	Tyrosine-protein kinase Wze	0.4555	1	28.57
Cps2F	SSU05_0569	Rhamnosyltransferase	1.5931	1	10.28
Cps2P	SSU05_0578	Sialic acid synthase	1.4255	6	76.63
Cps2Q	SSU05_0579	UNP-*N*-acetyglucosamine 2-epimerase	1.2001	17	53.58
Cps2R	SSU05_0580	Acetyltransferase	1.2131	2	44.23
Cps2S	SSU05_0581	CMP-*N*-acetylneuraminic acid synthetase	1.2388	2	59.47
**VIRULENCE-ASSOCIATED PROTEINS**					
ArcA	SSU05_0624	Arginine deiminase	0.4071	15	43.77
ArcB	SSU05_0626	Ornithine carbamoyltransferase	0.4561	10	35.91
ArcC	SSU05_0627	Carbamate kinase	0.3703	4	23.17
DltA	SSU05_0638	D-alanine-poly(phosphoribitol) ligase subunit 1	0.8186	11	25.24
Enolase	SSU05_1503	Phosphopyruvate hydratase	0.8146	26	72.64
GAPDH	SSU05_0155	Glyceraldehyde-3-phosphate dehydrogenase	0.7700	3	90.77
GlnA	SSU05_0160	Glutamine synthetase	0.8024	15	48.21
GtfA	SSU05_1555	Glycosidase	0.6384	2	6.22
IMPDH	SSU05_2183	Inosine 5′-monophosphate dehydrogenase	0.6649	1	45.60
PurA	SSU05_1966	Adenylosuccinate synthase	0.7722	22	60.23
SadP	SSU05_0272	Translation initiation factor 2 GTPase	0.6302	9	18.43
Sly	SSU05_1403	Suilysin	0.6552	4	11.26

#### Proteins involved in CPS synthesis

Except for Cps2C, the five enzymes involved in CPS synthesis were up-regulated in the mutant strain (Table 3). The induced enzymes include rhamnosyltransferase Cps2F, sialic acid synthase Cps2P, UNP-*N*-acetyglucosamine 2-epimerase Cps2Q, acetyltransferase Cps2R, and CMP-*N*-acetylneuraminic acid synthetase Cps2S.

#### Proteins involved in virulence

Several virulence factors were down-regulated in the mutant strain (Table 3). These factors include Sly, enolase, GAPDH, ADS (ArcABC), d-alanine-poly(phosphoribitol) ligase subunit 1 (DltA), glutamine synthetase (GlnA), glycosidase (GtfA), inosine 5′- monophosphate dehydrogenase (IMPDH), adenylosuccinate synthase (PurA), and translation initiation factor 2 GTPase (SadP).

## Discussion

The tRNA modification enzyme GidA contributes to proper folding and stability of tRNA and to the correct interaction between codon and anticodon during translation in eukaryotes and prokaryotes (Fislage et al., 2014). GidA acts as a regulator for protein expression either by its direct effects on translation efficiency of particular gene products or through its broader effects transmitted via expression of regulators (Kinscherf and Willis, 2002). In several pathogenic bacteria, GidA is considered to play roles in many particular cellular processes such as growth, cell division, and virulence regulation (Shippy et al., 2011). However, its functions are not always the same in different bacterial species. *S. suis* is an important zoonotic pathogen, and the role of GidA in *S. suis* is unclear. Our study demonstrated that GidA could regulate not only growth, cell division, and capsule synthesis but also virulence of this important pathogen.

First, a *gidA* deletion mutant was constructed. We found that the mutant Δ*gidA* grew much slower than the parental strain SC-19 on the TSA plate, and the size of the colonies of Δ*gidA* were obviously smaller than those of SC-19. The growth curves also confirmed the slow growth rate of the mutant strain. These results indicated that GidA can regulate *S. suis* growth. This finding agrees with the previous reports on *E. coli* (Von Meyenburg et al., 1982) and *S. enterica* (Rehl et al., 2013). To further understand the reason behind growth regulation, we performed a proteomics study. Numerous growth- and cell division-associated proteins were down-regulated by *gidA* disruption (Table 3). These proteins are classified into two classes: (i) DNA replication-, recombination-, and repair -related proteins, such as DNA primase (RnmV), DNA gyrase (GyrA and GyrB), DNA/RNA helicase (PcrA), site-specific recombinases (XerS and XerD), DNA repair ATPase (RecN), DNA recombination protein (RmuC), ribonucleases (RnhB, RNase H, G, and E); (ii) cell division-related proteins, including DivIVA, FtsQ, FtsI, StpK, PhpP, Cps2C, and MurD, which positively regulate cell division (Dinardo et al., 1982; Edwards and Errington, 1997; Ferreira et al., 2003; Janto et al., 2011; Šink et al., 2013; Fleurie et al., 2014; Zhu et al., 2014; Ahmed et al., 2015; Tsang and Bernhardt, 2015). These findings can explain the mechanism by which *gidA* disruption inhibits *S. suis* growth. However, two cell division-associated proteins, GpsB and FtsX, were up-regulated. GpsB is a negative regulator of cell division by interacting with DivIVA (Fleurie et al., 2014). Thus, GpsB up-regulation can inhibit cell division of *S. suis*. FtsX together with FtsE forms a dimer that act as an ABC transporter (Schmidt et al., 2004). The FtsEX protein complex plays a major role in regulating peptidoglycan hydrolases in response to signals from cell division (Sham et al., 2013), although the precise role of FtsX in coordinating peptidoglycan hydrolases remains unknown.

*gidA* deletion significantly reduced *S. suis* virulence. *In vivo* and *ex vivo* studies revealed that the mutant strain displayed reduced mortality and bacterial loads in mice, reduced ability to adhere to and invade in epithelial cells, and increased sensitivity to phagocytosis. This finding is also consistent with the regulation of virulence in *A. hydrophila* (Sha et al., 2004), *S. enterica* (Rehl et al., 2013), and *P. syringae* (Kinscherf and Willis, 2002). The virulence attenuation in *S. suis* by *gidA* disruption can be explained by the down-regulation of some virulence factors, including Sly, enolase, GAPDH, ADS (ArcABC), DltA, and SadP (Table 3). Sly is one of the most important virulence factors in *S. suis*, and contributes to pathogen's hemolytic activity, adhesion to and invasion of epithelial cells, host colonization, and ability to cross blood-brain barrier (Charland et al., 2000; Allen et al., 2001). The reduced hemolytic activity of Δ*gidA* was confirmed by the hemolysin assay (Figure 1D). The ADS encoded by the operon *arcABC* is also involved in ability of *S. suis* to adhere to and invade in epithelial cells (Degnan et al., 2000; Fulde et al., 2014), and to resist oxygen depletion, nutrient starvation, and acidic environments (Gruening et al., 2006). DltA is involved in d-alanylation of lipoteichoic acid which contributes to the survival of *S.suis* (Fittipaldi et al., 2008). In addition, enolase, GAPDH, and SadP are three important adhesins in *S. suis* (Ge et al., 2004).

Interestingly, the capsule of Δ*gidA* became much thicker than that of SC-19 (Figure 1C). This phenotype is consistent with the up-regulation of the enzymes, including Cps2F, Cps2P, Cps2Q, Cps2R, and Cps2S, which are involved in CPS synthesis (Table 3). In this study, only the tyrosine-protein kinase Cps2C was down-regulated. The orthologous protein of Cps2C in *Streptococcus pneumoniae* (CpsD) is a negative regulator for CPS production (Morona et al., 2003). To our best knowledge, this work is the first to report that CPS synthesis can be regulated by GidA in *S. suis*. CPS is considered a virulence factor in many bacteria, including *S. suis* (Feng et al., 2012). However, a report has suggested that the capsule only slightly contributes to the virulence of *S. suis* because both the virulent and avirulent strains can be fully encapsulated (Smith et al., 1999). Although the capsule of Δ*gidA* became thicker than that of the parental strain, the mutant strain was attenuated, and become more sensitive to phagocytosis. This finding is possibly affected by the use of multiple virulence factors in determination of *S. suis* virulence.

There are two technical issues needed to be discussed here. The first one is about the complementary strain. We failed to create a complementary strain of the mutant (in most cases it is difficult for *S. suis*). Therefore we have performed the RT-PCR to exclude polarity effect. In addition, identical phenotypes were observed with independently-obtained *gidA* mutants. In our view, this fact is sufficient to rule out the remaining possibility of distant secondary mutations whose chance to occur at the same place in two independent *gidA* mutants is extremely low. The second issue is about iTRAQ. Concerning that GidA can affect the accuracy of protein translation, truncated, or mistranslated proteins may be produced in the *gidA* mutant. These proteins may not be detected by iTRAQ. The information of this part of proteins was ignored in this study.

In conclusion, our data suggest that the tRNA modification enzyme GidA is a translational regulator for the expression of particular proteins involved in the growth, cell division, capsule synthesis, and virulence of the zoonotic *S. suis*. These findings provide a new insight that lead to our better understanding of the regulatory function of GidA in bacterial pathogens.

## Author contributions

The experiments were performed mainly by TG, MT, and WL, and some experiments were performed with the assistance of CZ, JZ, and LZ. TG, LL, and TZ analyzed the data. The study was designed by RZ. TG and RZ wrote the manuscript.

### Conflict of interest statement

The authors declare that the research was conducted in the absence of any commercial or financial relationships that could be construed as a potential conflict of interest.
